# New findings in preventing recurrence and improving renal function in AHUS patients after renal transplantation treated with eculizumab: a systemic review and meta-analyses

**DOI:** 10.1080/0886022X.2023.2231264

**Published:** 2023-08-10

**Authors:** Zhen Chun Tang, Huang Hui, Chunru Shi, Xiangmei Chen

**Affiliations:** a Guangdong Pharmaceutical University, Guangzhou, China; b Renal Medicine Department, Chinese PLA General Hospital, Beijing, China

## Abstract

**Background:**

The long-term mortality of kidney transplantation patients with atypical hemolytic uremic syndrome remains high, and the efficacy of the main treatment eculizumab is still controversial.

**Objective:**

A comprehensive systematic review and meta-analysis of clinical trials using eculizumab in renal transplant patients with atypical hemolytic uremic syndrome was conducted to evaluate the efficacy of this therapy and its impact on renal function.

**Methods:**

A comprehensive systematic search was conducted across multiple reputable databases, including Ovid (MEDLINE, EMBASE), PubMed, and the Cochrane Library (since database inception), to identify relevant studies exploring the use of eculizumab in patients with atypical hemolytic uremic kidney transplantation. Various renal function parameters, such as dialysis, rejection, glomerular filtration rate, serum creatinine, lactate dehydrogenase, and platelet count, along with patient relapse rates, were extracted and summarized using a combination of robust statistical methods, including fixed effects, random effects, and general inverse variance methods.

**Result:**

Eighteen trials with 618 subjects were analyzed. Our analysis suggests that the use of eculizumab is associated with a reduced likelihood of AHUS recurrence (odds ratio (OR) = 0.05, 95% CI: 0.00–0.13), as well as a significant reduction in the need for dialysis (odds ratio (OR) = 0.13, 95% CI: 0.01–0.32). Additionally, eculizumab treatment led to lower serum creatinine levels (mean differences (MD) = 126.931μmoI/L, 95% CI: 115.572μmoI/L–138.290μmoI/L) and an improved glomerular filtration rate (mean differences (MD) = 59.571 ml/min, 95% CI: 57.876 ml/min–61.266 mL/min). Our results also indicate that the use of eculizumab reduces the likelihood of rejection (odds ratio (OR) = 0.09, 95% CI: 0.01–0.22). Furthermore, the drug was effective in improving platelet counts (×10∧9/L) (mean differences (MD) = 163.421, 95% CI: 46.998–279.844) and lactate dehydrogenase levels (mean differences (MD) = 336.608 U/L, 95% CI: 164.816 U/L–508.399 U/L).

**Conclusions:**

Based on the meta-analysis, treatment with eculizumab can reduce dialysis rates and improve patients’ quality of life by enhancing renal function.

## 1. Introduction

Atypical hemolytic uremic syndrome (AHUS) is a rare, chronic multisystem disease that is life-threatening. The latest (2020) systematic review is the first to report the epidemiology of AHUS. The incidence of AHUS in the 20-year age group ranges from 0.26 to 0.75 per million population per year and 0.23 to 1.9 per million population for all age groups [1]. In the past, the prognosis of patients with AHUS was poor. Prior to the introduction of eculizumab, a majority of patients (over 50%) with this condition developed end-stage renal disease or succumbed to the illness within a year of initial diagnosis [2]. Inadequate complement regulation is thought to be a secondary cause of AHUS, and plasma therapy was once considered a potential contributing factor [3–7]. Although plasma infusion (PI) and plasma exchange (PEX) are sometimes used to treat AHUS, their efficacy is limited, and they may not prevent progression to end-stage renal disease [8]. Kidney transplantation therapy for AHUS patients is also not successful due to the high recurrence rate, especially for patients with complement Factor H (CFH) mutations. Bresin et al. reported a poor relapse rate of 85.7% after 1 year of organ transplantation [9], and the British and Italian registries reported almost equally poor results [10,11].

Eculizumab is a fully humanized monoclonal antibody whose therapeutic properties are to block terminal complement to inhibit uncontrolled complement activation. The emergence of eculizumab changes the situation in which AHUS patients can only face adverse results after kidney transplantation, which is also the preferred intervention to treat AHUS and prevent recurrence [12]. Eculizumab is highly effective at reducing mortality and recurrence rates in patients with chronic, severe AHUS [13]. Treatment with eculizumab has been shown to improve health-related quality of life by increasing the glomerular filtration rate (eGFR) [14]. A decrease in creatinine often eliminates the need for dialysis. While eculizumab is generally considered safe and well tolerated, its potential side effects remain a topic of controversy. While reports of adverse events associated with the drug are rare, the possibility of such events should not be overlooked, particularly in light of the increased susceptibility to certain enveloped bacteria, particularly meningococci, resulting from complement system blockade. In some cases, fulminant meningococcal disease has been reported in patients receiving eculizumab. Furthermore, the high cost of the drug is the largest current concern, limiting availability in many countries, costing millions of euros per patient per year. Moreover, it remains unclear whether eculizumab should be administered for life, and as such, many studies have proposed restrictive use strategies [15–17]. Guidelines exist for dosing eculizumab in AHUS patients, but there is limited clinical evidence to guide the optimal dosing strategy for individual patients. Although eculizumab is effective at reducing relapse rates in AHUS patients, it is expensive and may increase the risk of infection or rejection, which can lead to transplant failure.

Suarez et al. [18] suggested that eculizumab therapy might play a role in the prevention and treatment of recurrent AHUS. This study updated the prognostic indicators of AHUS patients. In addition to the most important effect of avoiding relapse, the drug improved patients’ hematological level and renal function. The analysis conducted by Suarez et al. [18] provides new insights into the use of eculizumab in AHUS patients after kidney transplantation.

## 2. Methods

### 2.1. Search strategy

**Figure 1.**
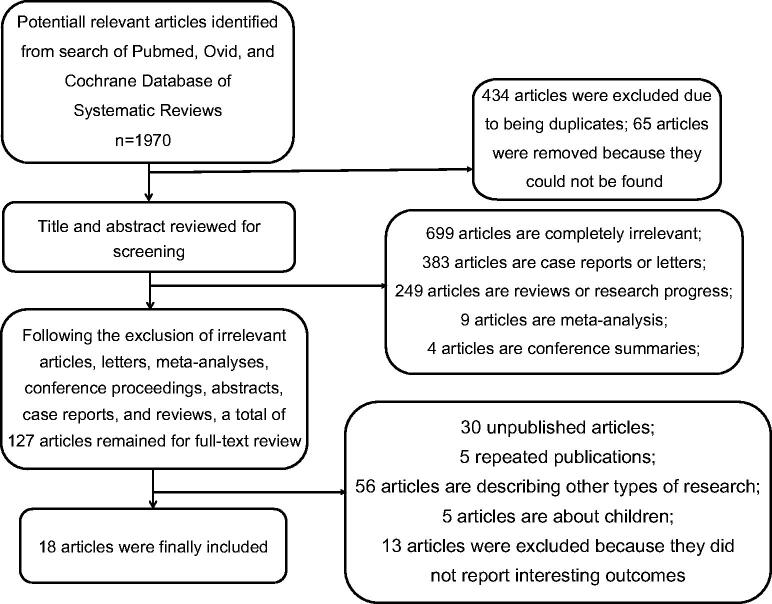
Detailed retrieval strategy. Forest map (control).

The systematic review protocol has been registered with PROSPERO (registration number was CRD42022360127, https://www.crd.york.ac.uk/PROSPERO/). A systematic literature search was conducted in PubMed, Ovid (MEDLINE, EMBASE databases) and the Cochrane systematic review database. The searched keywords were ‘kidney’, ‘transplant’, and ‘eculizumab’, which included their medical subject headings (MeSH) and free words. The search covered articles from October 1970 to 12 February 2023. A manual literature search was conducted for studies, including references to potentially relevant articles. This study was conducted according to the PRISMA (Preferred Reporting Item for Systematic Reviews and Meta-Analyses) statement. The detailed retrieval strategy is shown in Figure 1.

### 2.2. Selection criteria

1. Studies that met the following criteria were clinical trials or observational studies (cohort, case series, or cross-sectional studies) reporting the use of eculizumab to prevent and treat recurrence of AHUS after kidney transplantation;
2. The subjects were adults (aged 18 years or older) who had at least one kidney transplant or were receiving a kidney transplant;
3. The required outcome data must be provided, including whether the patient had AHUS recurrence, rejection, dialysis, serum creatinine (SCR), eGFR, lactate dehydrogenase (LDH), and platelet count (PLT).
4. The types of articles included case reports and studies, conferences, abstracts, literature reviews, meta-analyses, case reports and case series of patients treated with eculizumab.

Two authors (Tang and Yang) independently reviewed the articles for eligibility. We used kappa to test the consistency. As shown in Table 1, the kappa was 0.968 (≥0.75), which indicated that the two review results had good consistency. If there was any dispute, we discussed with the third author (Huang) to reach a common consensus. Inclusion was not limited by the size of the study.

**Table 1.** Kappa.

Symmetrical measurement		Value	Asymptotic standard error^a^	Approximate *T*^b^	Progressive significance
Protocol measurement	Kappa	.968	.031	10.918	.000
valid cases		127			

^a^Since a is constant, no statistics are calculated.

^b^Original assumptions are not assumed.

### 2.3. Data extraction

We extracted relevant data from two researchers (Tang and Yang). If there was any discrepancy, the third researcher (Huang) discussed and resolved the discrepancy in eligibility. The data were summarized using a structured information collection form. The following information was obtained from each study: the first author’s name, year of publication, sex (trial and control), demographics of kidney transplant patients, eculizumab regimen, and postrenal transplant outcomes (AHUS recurrence, rejection, dialysis, renal recovery, and thrombotic microangiopathy (TMA) events).

### 2.4. Statistical analysis

Stata SE15.1 and RevMan5.4 were used for data analysis, and statistical analysis was performed for all articles with the same indicators as much as possible to reduce bias. When a trial included multiple trial groups, only the relevant groups were included. We analyzed dichotomous data as odds ratios and/or continuous data as the mean differences (MDs) or standardized mean differences (SMDs), with a confidence interval (CI) of 95%. The results were pooled using Mantel–Haenszel odds ratios or mean differences (inverse variance). Between-study heterogeneity was assessed using Cochran’s sq test and the *I^2^* statistic (0 < *I^2^* < 25% indicated insignificant heterogeneity, 26% < *I^2^* < 50% indicated low heterogeneity, 51 < *I^2^* < 75% indicated moderate heterogeneity, and 76% < *I^2^* < 100% indicated high heterogeneity) [19], and fixed-effects models were used to evaluate interstudy heterogeneity. When moderate or large heterogeneity was identified, sensitivity analysis was performed using a random effects model due to the possibility of between-study variance. *p* < 0.05 (two-tailed test) was considered statistically significant. We conducted sensitivity analysis on some main outcomes. The presence of publication bias was assessed by subjective examination of funnel plots and Egger’s test [20].

## 3. Risk of bias within studies

The adapted Newcastle–Ottawa Scale (NOS) [21,22] was used to assess the quality of the included studies in this systematic review. The NOS consists of three columns: research population selection, intergroup comparability, and outcome measurement. It includes eight scoring items and nine scoring points. The evaluation focuses on whether the research design is reasonable, the clarity of the inclusion and exclusion criteria for the research objects, the clarity of the evaluation and diagnosis methods of cognitive impairment, and the correctness of the statistical methods. The highest score was 9, and four of the included studies were of high quality. By scoring the studies, the quality of the included literature can be judged to improve the credibility of this systematic review. For the remaining 10 case series, the study quality was assessed using the evaluation tool developed by the Australian JBI Evidence-Based Health Care Center in 2016 [23]. This tool consists of 10 evaluation items, each worth 1 point, for a total score of 10 points. Inclusion in the analysis required a score of 5 or above. The scores of the 18 studies included in this article are presented in Table 2.

**Table 2.** Characteristics of the included studies (demography).

Included in the study (Author and year)	Sample		Sex (man/female)		note	Nos score	JBI score
	experience	control	experience	control			
Glover 2022 [43]	35	23			Retrospective case note review	6	
Iraola 2022 [44]	53		(16/37)		Retrospective cohort study		8
Palma 2021 [27]	6		(0/6)		Retrospective case series		5
Nga 2021 [31]	27	11	(9/18)	(5/6)	Retrospective cohort study	6	
Portoles 2021 [45]	30	4	(16/14)	(3/1)	Retrospective cohort study	6	
Duineveld 2021 [46]	7		(1/6)		Retrospective study		6
Ardissino 2021 [39]	18	3	(5/13)	(1/2)	Case series study	5	
Gonzalez 2021 [47]	6		(3/3)		Case series		6
Kant 2020 [28]	10	9	(2/8)	(2/7)	Retrospective analysis	6	
Alpay 2019 [48]	8		(6/2)		Case series study		6
Duineveld 2017 [49]	1	16	(0/1)	(11/5)	Case series	4	
Andrade 2017 [29]	6		(3/3)		Case series		6
Manani 2017 [50]	3	1	(1/2)	(1/0)	Case series	4	
Legendre 2017 [51]	26		(10/16)		Case series		7
Levi 2017 [30]	12		(6/6)		Retrospective observational study		8
Mallett 2015 [33]	4		(0/4)		Retrospective cohort study		5
Matar 2014 [38]	7	5	(2/5)	(2/3)	Retrospective study	4	
Zuber 2012 [52]	14				Retrospective multicenter study		5

## 4. Result

Using the retrieval strategy of Figure 1, the two authors read the full text independently and included 18 studies according to the predetermined criteria; these studies were included in the present meta-analysis (Tables 2 and 3). The agreement of the two reviewers was very good for study selection.

**Table 3.** Characteristics of the included studies.

Included in the study (Author and year)	Interventions (Eculizumab)		Outcome
Glover 2022 [22]	Prevention: 900 mg, then 1200 mg		AHUS recurrence, dialysis, Serum creatinine, eGFR, rejection
Iraola 2022 [43]	/		AHUS recurrence, dialysis, Serum creatinine, eGFR, LDH, PLT
Palma 2021 [31]	/		AHUS recurrence, dialysis
Nga 2021 [34]	Treatment: 900 mg, 1200 mg	Prevention: 900 mg, and 1200 mg	AHUS recurrence, rejection, dialysis
Portoles 2021 [45]	900 mg, then 1200 mg		AHUS recurrence, dialysis, Serum creatinine
Duineveld 2021 [46]	900 mg, 1200 mg		AHUS recurrence, Serum creatinine
Ardissino 2021 [39]	customized		AHUS recurrence, dialysis, rejection, Serum creatinine
Gonzalez 2021 [47]	1200 mg then 900 mg/week 4 times, then 1200 mg		AHUS recurrence, rejection, dialysis, Serum creatinine
Kant 2020 [28]	Treatment: 900 mg, then 1200 mg	Prevention:1200 mg, 900 mg,1200 mg	AHUS recurrence, dialysis, Serum creatinine, eGFR
Alpay 2019 [48]	900 mg, 1200 mg		AHUS recurrence, rejection, Serum creatinine, LDH
Duineveld 2017 [49]	/		AHUS recurrence, rejection, Serum creatinine, eGFR
Andrade 2017 [29]	Treatment: 900 mg; 1200 mg,	Prevention: 900 mg, 1200 mg	AHUS recurrence, Serum creatinine, eGFR, LDH, PLT
Manani 2017 [50]	customized		AHUS recurrence, dialysis, rejection
Legendre 2017 [51]	900 mg, 1200 mg		AHUS recurrence, rejection, dialysis, eGFR, LDH, PLT
Levi 2017 [30]	Treatment: 900 mg, then 1200 mg	Prevention: 1200 mg, 900 mg. Then 1200 mg	AHUS recurrence, rejection, dialysis, Serum creatinine, eGFR
Mallett 2015 [33]	900mg, 1200 mg		AHUS recurrence, rejection, Serum creatinine, LDH, PLT
Matar 2014 [38]	/		AHUS recurrence, dialysis, Serum creatinine, eGFR
Zuber 2012 [52]	/		AHUS recurrence

*Note*. eGFR: glomerular filtration ratep; LDH: lactate dehydrogenase; PLT: platelet count.

Eighteen studies with a total of 618 patients were included in this study. We set 7 outcome indicators, including AHUS recurrence, rejection, dialysis, and follow-up patients’ eGFR, LDH, PLT, and SCR levels in 3 studies with serum creatinine markers and five studies with AHUS recurrence markers and 5 studies with dialysis markers, all had a control group (no treatment with eculizumab). We separated these studies from the results and analyzed them. In addition, we used AHUS recurrence and whether the patient was finally on dialysis, as well as the patient’s eGFR and creatinine levels, as the main indicators. Because preventing AHUS recurrence was the main task of eculizumab and the purpose of this study, serum creatinine was the current gold standard for measuring changes in renal function and identifying AKI [24], while the decline in glomerular filtration rate (eGFR) can directly reflect the degree of renal failure [25]. The rejection and the level of LDH and PLT in patients were secondary indicators because they can indicate TMA response [26] (normalization of platelet count, recovery of renal function, reduction of serum creatinine ≥25%) and hematological response (platelet count, lactate dehydrogenase).

### 4.1. AHUS recurrence

The present study aimed to investigate the effectiveness of eculizumab in preventing the recurrence of AHUS in patients who had undergone kidney transplantation. A total of 12 studies, with all assessing AHUS recurrence but 12 without a control group, were included in this meta-analysis. The results of the heterogeneity test indicated moderate heterogeneity among the studies (*I*^2^ = 58.61%, *p* < 0.1), prompting a sensitivity analysis to explore potential reasons for this finding. Fortunately, none of the studies interfered significantly with the results, indicating good stability of the study. Funnel plots further supported the reliability of the findings. A random-effect model was employed, and the combined effect size of the 12 studies was 0.05, with a 95% confidence interval of 0.00–0.13, suggesting that the use of eculizumab after kidney transplantation was effective in reducing the recurrence of AHUS. The results were statistically significant (*z* = 2.37, *p* ≤ 0.05) and have important clinical implications. A detailed forest map can be found in the attached table.

#### 4.1.2. Dialysis

In our meta-analysis of seven studies examining the effect of eculizumab on post-transplant dialysis rates, heterogeneity testing revealed significant heterogeneity among the studies (*I*^2^ = 85.19%, *p* < 0.1). To address this, a sensitivity analysis was conducted, and it was found that Palma’s research caused significant interference with our results [27]. Once this study was removed, the heterogeneity test yielded more favorable results (*I*^2^ = 59.48%, *p* < 0.1), indicating moderate heterogeneity. Using a random-effects model, our meta-analysis showed that eculizumab had a significant effect on reducing post-transplant dialysis rates, with an effect size of 0.13 and a 95% CI of 0.01–0.32 (*z* = 2.35, *p* ≤ 0.05). These findings have important clinical implications and suggest that eculizumab may be a promising therapeutic option for reducing post-transplant dialysis rates. Please refer to the attached table for detailed information on our results.

#### 4.1.3. Serum creatinine

In our meta-analysis of ten studies evaluating the effect of eculizumab on serum creatinine levels following renal transplantation, significant heterogeneity was observed in the literature (*I*^2^ = 84.3%, *p* < 0.1). To address this, we conducted a sensitivity analysis to investigate the reasons for the heterogeneity. Our analysis revealed that the studies of Emily and Jose, Alpay, and Mallet significantly interfered with our results. After removing these studies, the heterogeneity test was continued (*I*^2^ = 39.3%, *p* > 0.1), and the fixed effect model was employed for analysis.

The results of our meta-analysis based on the fixed effect model showed that the combined effect size of the remaining six studies was 126.931μmoI/L, with a 95% CI of 115.572μmoI/L–138.290μmoI/L, indicating statistical significance. Our findings (*z* = 15.1, *p* ≤ 0.05) suggest that the use of eculizumab can significantly reduce serum creatinine levels after renal transplantation, which has important clinical implications. Further details on our results can be found in the attached table.

#### 4.1.4. Glomerular filtration rate

The six pieces of literature in this study evaluated the glomerular filtration rate level of the patients. The results of the heterogeneity test showed no significant heterogeneity among the studies (*I*^2^ = 43.8%, *p* > 0.1), and a fixed-effects model was selected to ensure the accuracy and stability of the study.

The meta-analysis using a fixed-effects model showed a statistically significant improvement in eGFR after renal transplantation with eculizumab, with an aggregated effect size of 59.571 mL/min and a 95% CI of 57.876 mL/min–61.266 mL/min. The results (*z* = 17.19, *p* ≤ 0.05) have clinical significance, indicating that eculizumab improves eGFR after renal transplantation. These details are presented in the attached table.

### 4.2. Minor

#### 4.2.1. Rejection

The seven pieces of literature in this study included whether the patients experienced rejection. After the heterogeneity test, the results (*I*^2^ =56. 41% and *p* < 0.1) indicated that there was a moderate difference between the literature selected in this study. When the heterogeneity reached moderate heterogeneity, sensitivity analysis continued to investigate the reasons for the heterogeneity.

On the seven pieces of literature in this study, none of the literature caused great interference to the results of this meta-analysis, which meant that this study had good stability.

The meta-analysis based on the random effect model showed that the combined effect size of seeeven studies was 0.09, and the 95% CI was 0.01–0.22, which was statistically significant. The results (*z* = 2.46, *p* ≤ 0.05) indicate that the use of eculizumab reduces the likelihood of rejection reactions after kidney transplantation. Details are shown in the attached table.

#### 4.2.2. Platelet count

The four pieces of literature in this study evaluated the PLT of patients. After the heterogeneity test, the results (*I^2^* was 95.6% and *p* < 0.1) suggested that there was significant heterogeneity among the literature selected in this study. Therefore, sensitivity analysis was continued to investigate the reasons for the heterogeneity.

Sensitivity analysis was performed on the four pieces of literature in this study, and two pieces of literature interfered with the results of this meta-analysis. Since few studies include the assessment of PLT in patients, any literature that may cause heterogeneity will not be excluded and continue to be analyzed based on the random effect model. More studies will be included in the evaluation of this index in the future.

A meta-analysis based on the random effects model showed that the combined effect of 4 studies was 163.421/mm³, and the 95% CI was 46.998/mm³–279.844/mm³, with statistical significance. The results (*z* = 2.75 and *p* ≤ 0.05 (Q test) suggested that eculizumab was effective in improving platelet count after kidney transplantation, which has certain clinical significance. Details are shown in the attached table.

#### 4.2.3. Lactate dehydrogenase

This study analyzed five articles that evaluated LDH levels in patients after receiving eculizumab following renal transplantation. The heterogeneity test revealed significant differences among the selected studies (*I*^2^ = 94.0%, *p* < 0.1), and a random-effects model was selected to ensure the accuracy and stability of the study. Sensitivity analysis was conducted, and only one study interfered with the results, indicating good stability in this meta-analysis. Due to the limited number of studies evaluating LDH levels in patients, potentially heterogeneous literature was not excluded.

**Figure 2.**
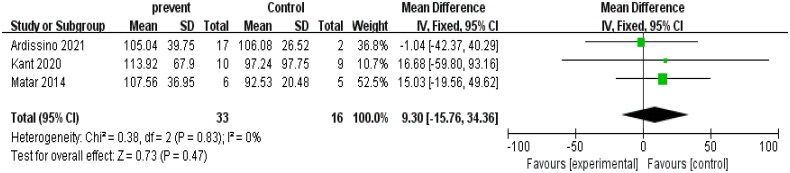
Meta-analysis of single-arm studies.

The meta-analysis using a random-effects model showed a statistically significant effect of eculizumab on LDH after renal transplantation, with a combined effect size of 336.608 U/L and a 95% CI of 164.816 U/L–508.399U/L. The results (*z* = 3.840, *p* ≤ 0.05) were clinically significant, indicating that eculizumab has a significant effect on LDH levels after renal transplantation. The details are presented in the attached table. All analyses conducted on studies without a control group will be summarized in Figure 2, which will provide a comprehensive summary of the results. The results of these studies will be analyzed and presented in a clear and concise manner to facilitate understanding and interpretation.

### 4.3. Studies with control groups

#### 4.3.1. Serum creatinine

All of the included studies in this analysis assessed serum creatinine levels, with three of them having a control group. The results of the heterogeneity test (*I*^2^ = 0, *p* > 0.1) indicated that there was no heterogeneity among the selected studies, making the fixed effects model suitable for meta-analysis.

Based on the fixed effects model, the meta-analysis of the three studies showed a combined effect size of 9.3μmoI/L with a 95% confidence interval ranging from −15.762μmoI/L to 34.358μmoI/L. Although statistically significant, the forest plot displayed no significant improvement in creatinine levels following the use of eculizumab after kidney transplantation (*z* = 0.73, *p* = 0.47).

**Figure 3.**
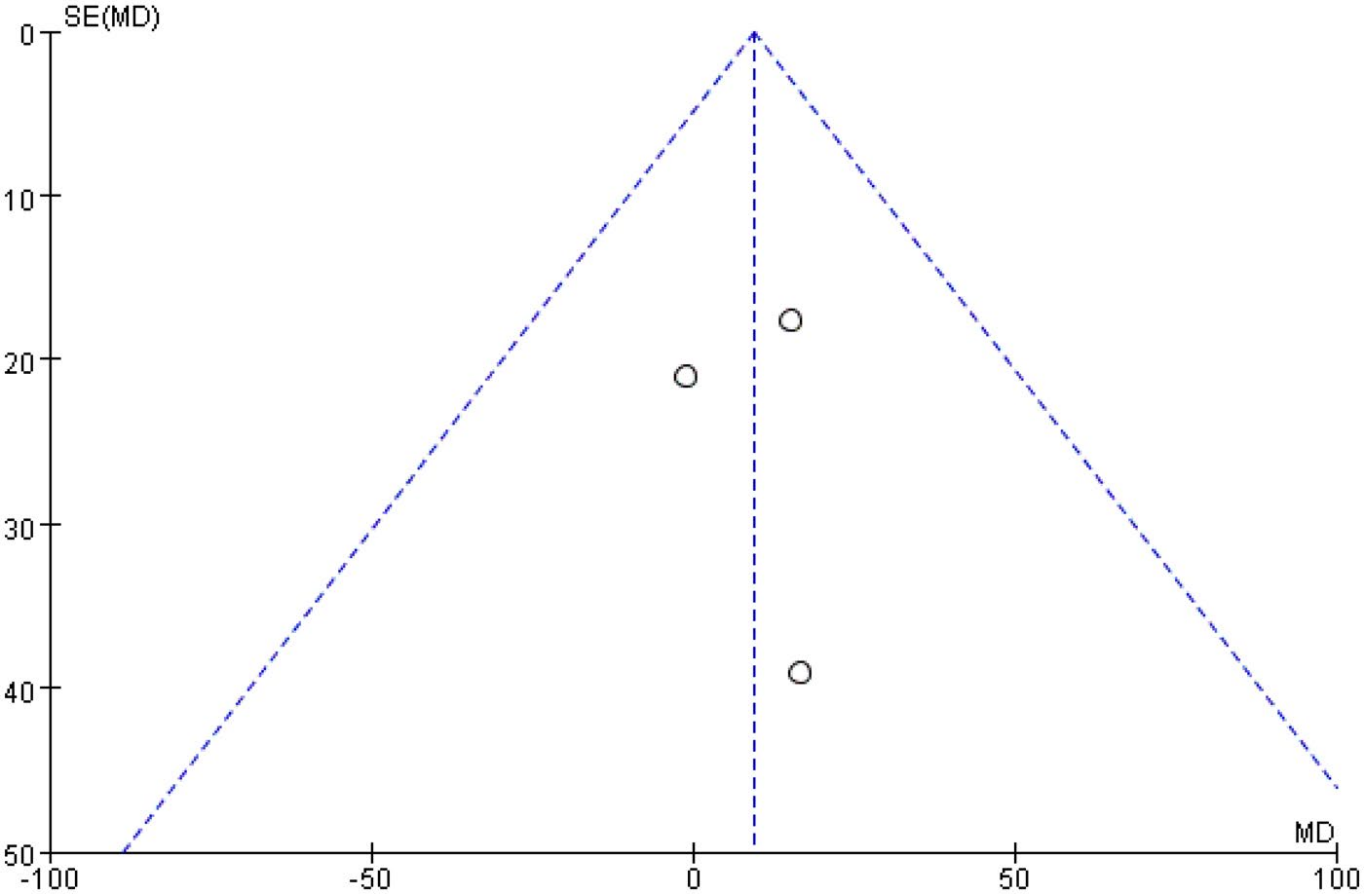
Serum creatinine.

**Figure 4.**
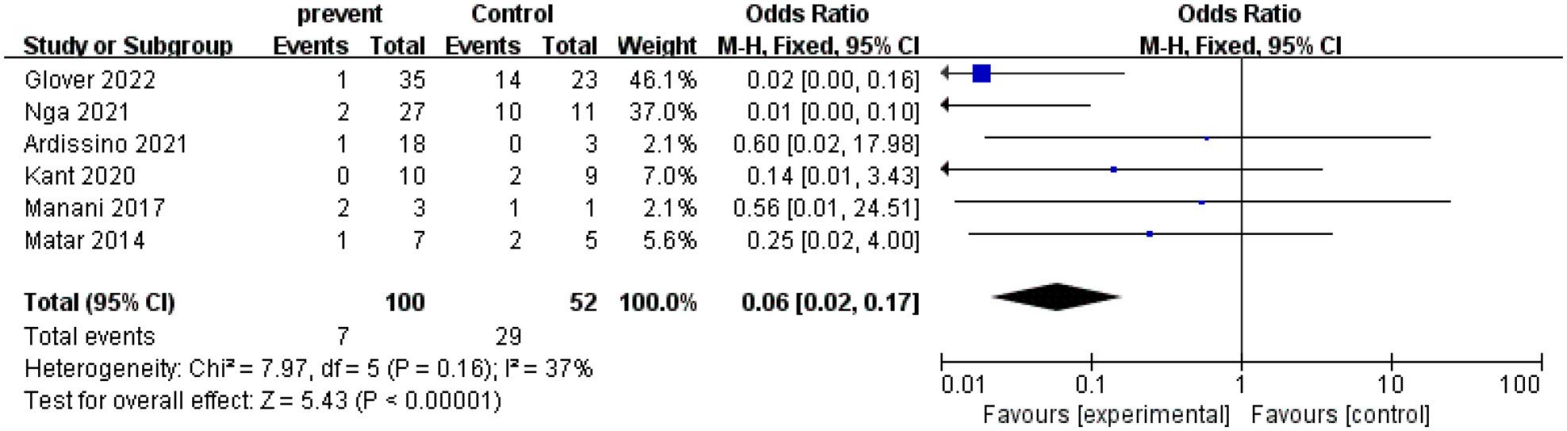
Funnel plot of serum creatinine.

In summary, the analysis results indicated that the use of eculizumab did not lead to an improvement in creatinine levels among postkidney transplant patients, as depicted in Figure 3 for the detailed forest plot and Figure 4 for the funnel plot. This finding could be attributed to the limited number of studies with a control group on creatinine levels (only three studies), which might have affected the statistical power of the analysis. Nonetheless, we have analyzed ten studies with statistically significant and credible results.

#### 4.3.2. AHUS recurrence

**Figure 5.**
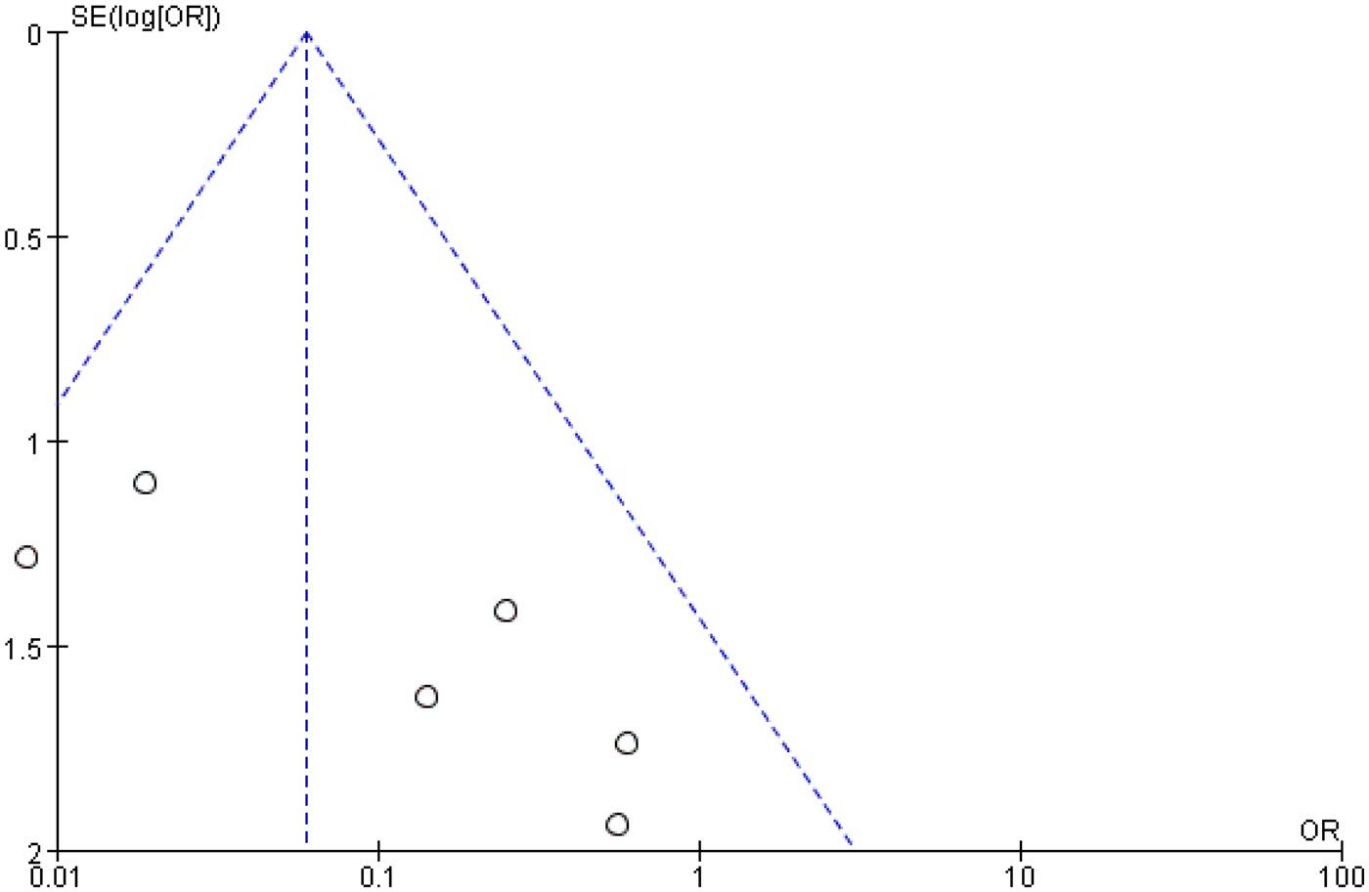
AHUS recurrence.

**Figure 6.**
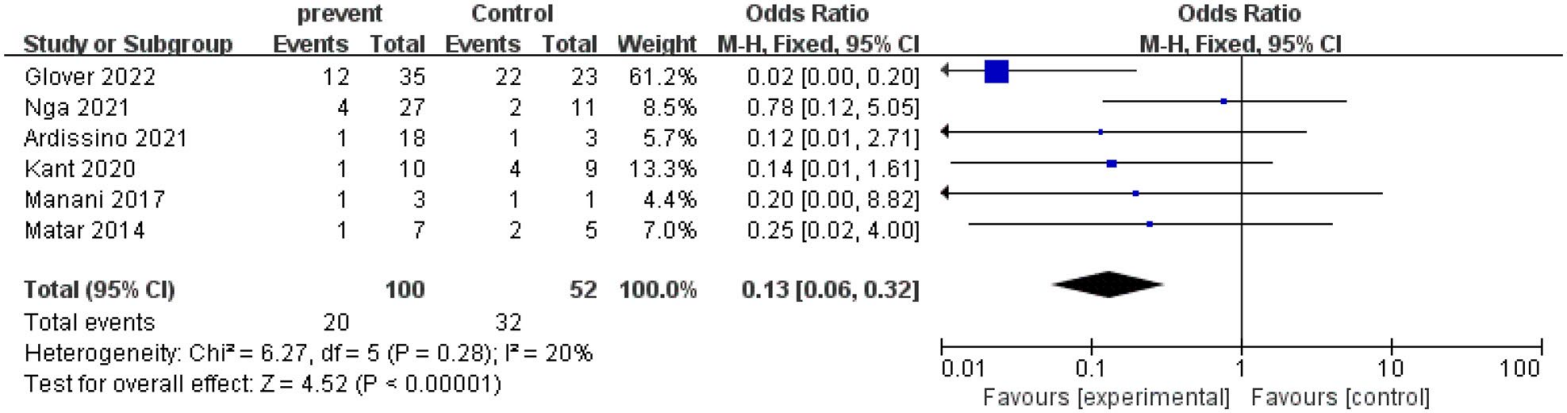
Funnel plot of AHUS recurrence.

This study, which included a control group, analyzed the efficacy of eculizumab in preventing the recurrence of AHUS in patients undergoing kidney transplantation. A total of 6 studies were included in the analysis. The heterogeneity test showed that the heterogeneity among studies was low (*I*^2^ = 37%, *p* > 0.1), indicating that the fixed effect model was suitable for analysis. The combined odds ratio (OR) value of the four studies was 0.06, and the 95% confidence interval (CI) was 0.02–0.17, indicating that the recurrence rate of AHUS was significantly reduced after eculizumab treatment (*Z* = 5.43, *p* < 0.00001). The forest plot presented in the study visually displays these results, which suggest that eculizumab may be an effective therapy for the prevention of AHUS recurrence after kidney transplantation. The detailed forest diagram can be seen in Figure 5, and the funnel diagram can be seen in Figure 6.

#### 4.3.3. Dialysis

**Figure 7.**
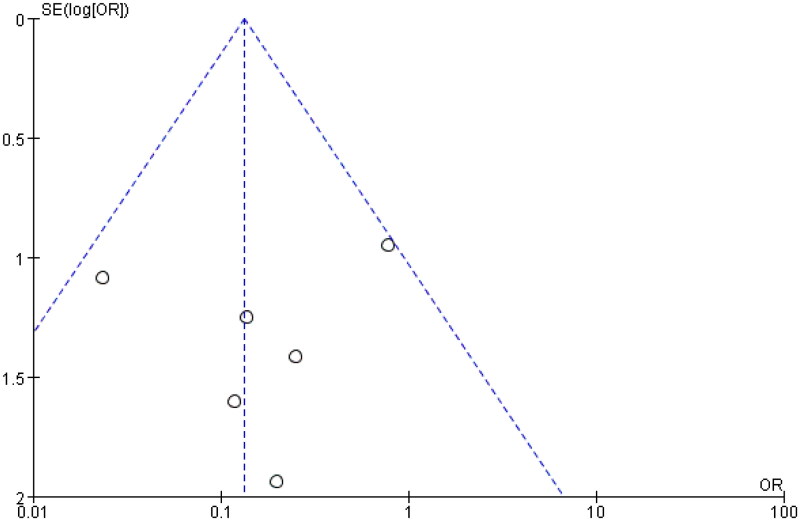
Dialysis.

**Figure 8.**
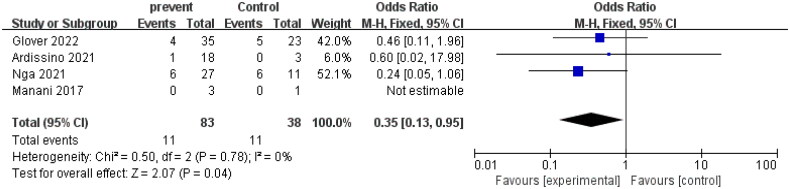
Funnel plot of dialysis.

The study analyzed data from six control groups to evaluate the effect of eculizumab treatment on dialysis rates in patients with AHUS undergoing kidney transplantation. The heterogeneity test indicated no significant heterogeneity among studies (*I*^2^ = 20%, *p* = 0.28 > 0.1), suggesting that a fixed effect model was appropriate. The combined odds ratio (OR) of the six studies was 0.13 with a 95% confidence interval (CI) of 0.06–0.32, indicating a significant reduction in dialysis rates with eculizumab treatment compared to controls (*Z* = 4.52, *p* < 0.00001). The forest plot presented in the study visually displays these results. The funnel plot, which was generated to assess publication bias, showed no significant asymmetry, suggesting no evidence of publication bias in the literature. These findings imply that eculizumab could be an effective therapy for preventing dialysis in AHUS patients undergoing kidney transplantation. The detailed forest diagram can be seen in Figure 7, and the funnel diagram can be seen in Figure 8.

#### 4.3.4. Rejection

**Figure 9.**
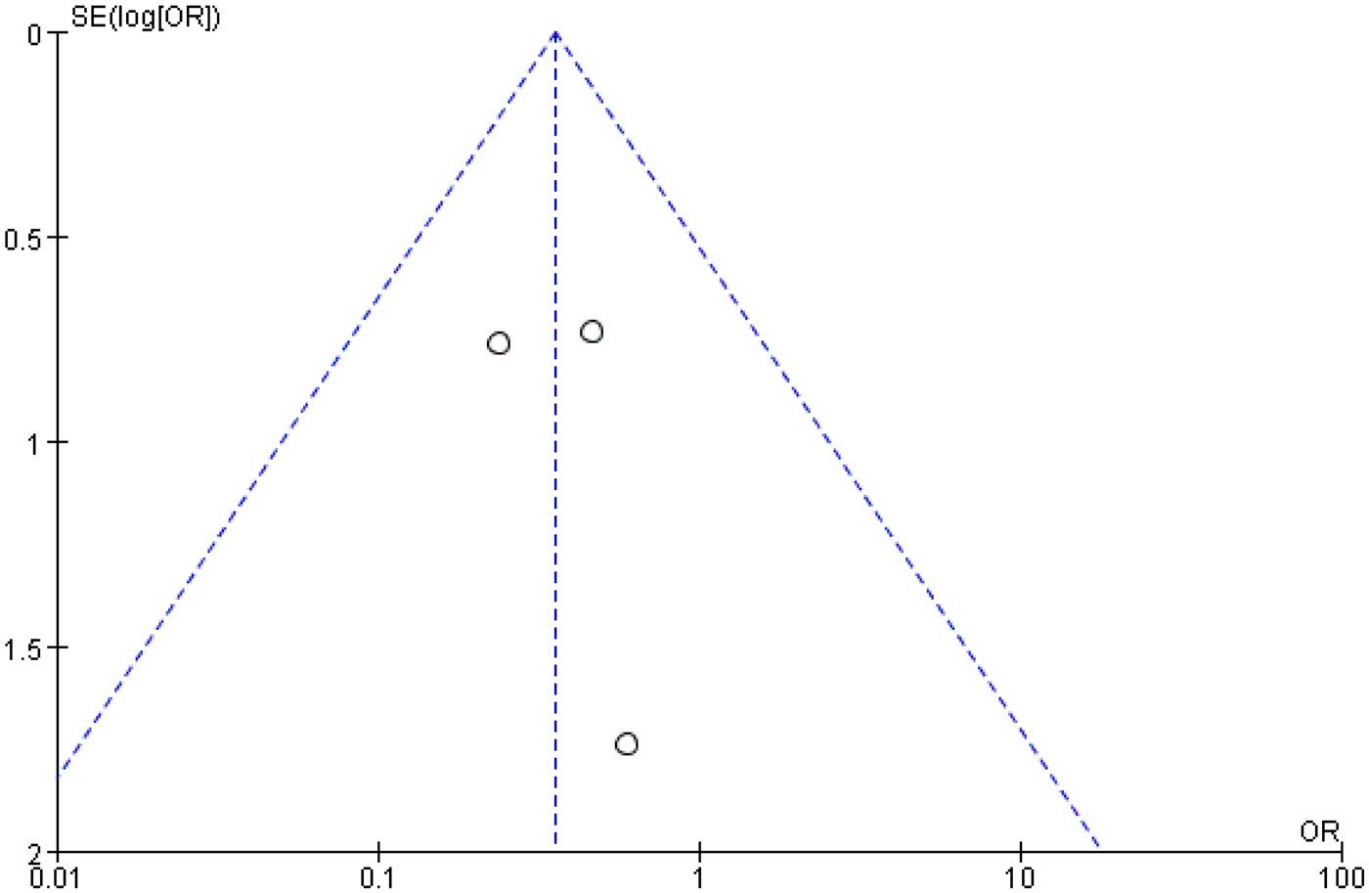
Rejection.

**Figure 10.**
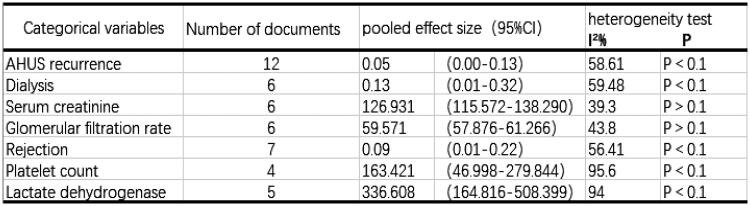
Funnel plot Rejection. Forest map (without control) appendix.

The aim of this study was to analyze the impact of eculizumab on the rejection of patients with AHUS after kidney transplantation. The study included four controlled trials. After conducting a heterogeneity test with *I*^2^ = 0% and *p* = 0.78 > 0.1, the fixed effect model was found to be suitable for analysis, and a forest plot was generated accordingly. The results indicated a pooled effect size of 0.35 and a 95% confidence interval of 0.13–0.95, demonstrating that the use of eculizumab significantly reduced the occurrence of rejection in AHUS patients after kidney transplantation (*Z* = 2.07, *p* = 0.04). These findings suggest that eculizumab may be an effective therapy in reducing rejection rates in this patient population. The detailed forest diagram can be seen in Figure 9, and the funnel diagram can be seen in Figure 10.

## 5. Offset evaluation

Bias assessment of some metrics, Begg’s rank correlation test, and Egger’s linear regression were used to assess publication bias. Two-sided *p* values (≤0.05) were considered to be significant.

Funnel plots were drawn to test whether publication bias existed in articles on the recurrence of AHUS in this study, and the symmetry of the funnel plot meant that there was no publication bias. The funnel plot of this study is shown in the appendix. The symmetry test (*p* ≥ 0.05) indicated that there was no publication bias in the literature of this study.

A funnel plot was drawn to test whether there was publication bias in the analysis of rejection (see the appendix for the funnel plot). The symmetry of the funnel plot means that there is no publication bias.

Furthermore, a symmetry test was performed on the graph (*p* ≥ 0.05), which indicated that there was no publication bias in the literature of this study.

The recurrence of AHUS (control group) had a funnel plot drawn to examine whether there was publication bias in the articles on the recurrence of AHUS. The symmetry of the funnel plot meant that there was no publication bias. The funnel plot of this study is shown in the attachment. Based on Begg’s bias test (*p* = 0.734), the four studies selected for this study had no publication bias.

However, due to the limited number of relevant articles in the relevant database, we will not evaluate the above remaining indicators (eGFR, SCR, LDH, PLT, dialysis status) for the time being. If more high-quality related articles are published in the future, the meta-analyses can be better summarized.

## 6. Discussion

Eculizumab remains the standard of care for preventing AHUS recurrence after kidney transplantation, especially in patients with confirmed or suspected genetic mutations in complement-regulating proteins. Other alternative methods include plasma exchange or infusion, which aims to remove or dilute the circulating autoantibodies or mutant complement proteins that cause AHUS. However, plasma therapy may not be effective in patients with genetic mutations in complement-regulating proteins, which account for approximately 60% of AHUS cases. Another alternative method is the use of other complement inhibitors, such as anti-C5a antibodies, anti-Factor B antibodies, or Factor H analogs. These agents target different components of the alternative complement pathway and may have different pharmacokinetics and safety profiles than eculizumab. However, these agents are still in the early stages of development and have not been tested in large clinical trials for AHUS patients undergoing kidney transplantation. Further studies are needed to evaluate the long-term efficacy and safety of eculizumab. This meta-analysis included evaluating multiple metrics to measure eculizumab and demonstrated that it produced meaningful efficacy in patients with AHUS after kidney transplantation.

This meta-analysis showed that eculizumab was effective in preventing AHUS recurrence, reducing dialysis rates, and improving renal function in AHUS patients after renal transplantation. In addition, a study by Kant, Luis Gustavo, Charlène Levi, and Hong Si Nga [28–31] modified the strategy of using eculizumab by setting a patient group for preventive therapy. Most transplant patients with a history of AHUS had a significantly lower recurrence rate after receiving eculizumab prophylaxis than those who did not receive eculizumab prophylaxis. Andrew et al. [32] divided patients in the global AHUS registry into prophylactic and post-transplant groups and found that patients who received eculizumab before transplantation had significantly better kidney function than post-transplant patients, while those who received dialysis had a significantly increased risk of dialysis after transplantation. These findings provide information on how to use eculizumab. However, some studies showed that early use of eculizumab did not help the kidneys of patients with AHUS [33], and their studies have certain limitations. When to discontinue the drug after use is also important in the eculizumab approach to evaluate AHUS recurrence [16,34]. Two studies mentioned that eculizumab was once recommended for lifelong treatment, but a prospective phase 4, multicenter, uncontrolled study provided evidence that once complete remission is achieved, most patients with AHUS are safe to discontinue eculizumab [17]. These studies show that the decision to discontinue eculizumab is based on the patient’s risk factors, such as the patient’s hematological parameters and renal function levels [35].

Three studies with control groups were included in our study, and the results of studies evaluating dialysis were consistent with those without control groups, so no further elaboration will be made. In a study evaluating the SCR level and AHUS recurrence in patients, opposite results were obtained. Eculizumab did not improve patients’ SCR levels well enough to prevent recurrence, which may be due to the control included in the study. The cohort was smaller, and other confounding factors were present; for example, the treatment cohort included patients with CFH mutations, who generally had a higher relapse rate [36,37], and the strategy of using eculizumab was different.

In one study evaluating creatinine levels [38], there were three patients in the treatment group who only used the 6-month treatment strategy, and their creatinine levels were significantly higher than those of patients who used the lifelong treatment strategy, even compared to patients who did not use the lifelong treatment strategy. The fact that an almost equal number of patients received eculizumab may have contributed to this finding. This was also the case in Sam’s study [28], where four out of nine trial groups had no lifetime treatment. In Gianluigi’s study [39], the sample size of the control group was only two. In the future, more prospective randomized controlled trials are expected to increase the sample size of this indicator and affect the results.

In the controlled study of Sam et al. [28] for the recurrence of AHUS, 40% of the patients in the experimental group carried the CFH gene, while only 30% carried the CFH gene in the control group. Although no patient in the experimental group relapsed, those in the control group relapsed. The rate was not high enough to be statistically significant. The CFH produced in this group was low, and these patients were also receiving maintenance therapy, such as prednisone and tacrolimus, to prevent rejection [40–42]. In the study by Gianluigi et al. [39], only 6 of the 21 patients did not have the CFH mutation gene, while 3 were controls who did not receive eculizumab and 1 was a control who received plasma prophylaxis (plasma prophylaxis PP).

Eculizumab can prevent or reverse thrombotic microangiopathy and improve kidney function in AHUS patients. However, eculizumab is also very expensive and has potential side effects. The cost of eculizumab treatment for AHUS patients can range from €40,241 to €50,794 per patient per month, depending on the treatment regimen and the recurrence rate. Eculizumab also increases the risk of serious infections such as meningococcal disease, so patients need to receive vaccinations and antibiotics before and during treatment.

Due to ongoing research in this field, there is a dearth of original studies in both Chinese and English databases, which is a limitation inherent in the current state of knowledge. Additionally, the potential influence of gene mutations cannot be ruled out, and while these may have minimal impact on the findings, the use of a control group remains necessary to mitigate potential sources of bias. Future research should aim to explore the specific role of eculizumab in the context of CFH gene disruption. Furthermore, the majority of the included studies in this area were of a nonrandomized and single-arm design, which led to an overall high risk of bias.

In general, our study showed that the use of eculizumab in patients with recurrence after renal transplantation has good efficacy and safety and can prevent AHUS recurrence by improving renal function and blood normalization without causing rejection.
